# Resilience Predicts Self-Stigma and Stigma Resistance in Stabilized Patients With Bipolar I Disorder

**DOI:** 10.3389/fpsyt.2021.678807

**Published:** 2021-05-21

**Authors:** Fabienne Post, Melanie Buchta, Georg Kemmler, Silvia Pardeller, Beatrice Frajo-Apor, Alex Hofer

**Affiliations:** Department of Psychiatry, Psychotherapy and Psychosomatics, Division of Psychiatry I, Medical University Innsbruck, Innsbruck, Austria

**Keywords:** bipolar disorder, resilience, stigma, premorbid functioning, psychopathology

## Abstract

The identification of factors that prevent self-stigma and on the other hand promote stigma resistance are of importance in the long-term management of bipolar disorder. Accordingly, the aim of the current study was to investigate the association of factors deemed relevant in this context, i.e., resilience, premorbid functioning, and residual mood symptoms, with self-stigma/stigma resistance. Sixty patients diagnosed with bipolar I disorder were recruited from a specialized outpatient clinic. Self-stigma and stigma resistance were measured by the Internalized Stigma of Mental Illness (ISMI) Scale. The presence and severity of symptoms were assessed by the Montgomery-Asberg Depression Rating Scale (MADRS) and the Young Mania Rating Scale (YMRS). Resilience and premorbid functioning were measured by the Resilience Scale (RS-25) and the Premorbid Adjustment Scale (PAS), respectively. Resilience correlated negatively with self-stigma and positively with stigma resistance and was a predictor for self-stigma/stigma resistance in multiple linear regression analysis. Residual depressive symptoms correlated positively with self-stigma and negatively with stigma resistance. There were no significant correlations between sociodemographic variables, premorbid functioning as well as residual manic symptoms and self-stigma/stigma resistance. The findings of this study implicate that resilience may be considered as an important component of self-stigma reduction interventions.

## Introduction

Patients suffering from bipolar disorder (BD) may encounter many difficulties during the course of the illness that can have a negative impact on the outcome. Not only do they face challenges that are associated with the symptoms of the illness itself and side effects of treatment, but stigmatizing attitudes can play a negative role on the course of the illness. All these factors can lead to deprivation of factors which define quality of life such as pursuing a good job, living in a safe environment, having satisfactory health care, and having a wide spectrum of social contacts (1).

Although there are efforts to reduce stigma, stigmatizing attitudes toward the mentally ill is still an issue across all levels of society (2). Consequently, it can lead to patients applying those negative stereotypes and stigmatizing attitudes toward themselves, which is known as self-stigma (3). Self-stigma may delay health seeking behavior and may also prevent effective treatment (4). However, it is important to note that not all patients experiencing public stigma automatically also suffer from self-stigma (5). Hence, self-stigma can be seen as a modifiable risk factor (6) and in this context, identifying factors that help prevent its development or identifying factors that promote stigma resistance can be of great importance in the long-term management of bipolar disorder. On the one hand, it may help us identify vulnerable patients, and on the other hand, it may facilitate the implementation of protective and therapeutic interventions. In this context, a study by Cuhadar and Cam (7) showed a positive effect of a psychoeducation program on levels of self-stigma in bipolar patients. In turn, previous research has shown that self-stigma has negative effects on self-esteem (8–11), quality of life (8, 11–14), treatment adherence (12) as well as socio-occupational participation and functioning (8, 15). However, to the best of our knowledge, the association between resilience and premorbid functioning on one hand and self-stigma on the other have not yet been investigated in bipolar disorder.

In the past years, the role of resilience in serious mental illnesses has become a topic of growing interest. In schizophrenia, it has been shown to have a positive impact on the long-term outcome (16) and high levels of resilience have been shown to reduce the risk of suicide in both schizophrenia (17) and depression (18). However, research on the role of resilience in patients suffering from bipolar disorder specifically, is rather scarce and its effects on the long-term outcome are yet to be investigated. In our recent studies in patients with bipolar disorder, we have shown resilience to be associated with quality of life (14, 19, 20), self-esteem, spirituality, and hopelessness (19). Lee et al. (21) have also shown a positive correlation between resilience and quality of life. A further study has shown that low levels of resilience were associated with high levels of impulsivity and an increased number of depressive episodes (22). These findings show that resilience can act as a protective factor and may have a positive impact on the long-term outcome. Resilience may also play a positive role in the degree of self-stigma a patient perceives and it may hence play a positive role in building stigma resistance, as we have recently shown in one of our recent studies investigating self-stigma in patients with schizophrenia (23).

In general, the role of premorbid functioning in bipolar disorder remains unclear. So far, there are only few studies to support the existence of premorbid disturbance in those affected and findings are inconsistent. Cannon et al. (24), for example, reported on significantly lower overall premorbid functioning levels in participants with adult-onset BD compared to healthy control subjects, and Gade et al. (25) showed that premorbid functioning is a notable predictor of overall functioning among adolescents and adults with BD. Still other investigations, in turn, have shown stable premorbid features in patients with BD (26, 27) and one cross-sectional study even identified higher premorbid functioning among patients with BD compared to healthy controls (28).

Due to the lack of studies regarding this topic in bipolar disorder we decided to investigate the associations of the above mentioned factors in stabilized outpatients and hypothesized that low resilience, poor premorbid functioning as well as more severe residual symptoms would be associated with low stigma resistance and high self-stigma.

## Materials and Methods

Sixty patients diagnosed with bipolar I disorder were recruited from a specialized outpatient clinic. Diagnosis was confirmed by the Mini International Neuropsychiatric Interview (M.I.N.I) (29). Study participants had to be native German speakers aged between 18 and 65 years and had to be clinically stable, i.e., they had not been admitted and had no alteration in psychopharmacological treatment in the last 6 months prior to study inclusion. Patients with a history of neurological and severe somatic illness, cerebrovascular dysfunction as well as patients with dementia were excluded from the study. The study was approved by the local ethics committee and patients had to provide written informed consent. All scales used in the study are validated German translations of the original scales.

### Psychopathology

In patients, the presence and severity of depression and mania were measured by the Montgomery-Åsberg Depression Rating Scale (MADRS) (30) and the Young Mania Rating Scale (YMRS) (31), respectively. The MADRS consists of 10 items (apparent sadness, reported sadness, inner tension, reduced sleep, reduced appetite, concentration difficulties, lassitude, inability to feel, pessimistic thoughts, and suicidal thoughts) and each item yields a score of 0 to 6. The overall score ranges from 0 to 60 with higher scores reflecting more severe depression. The YMRS consists of 11 items with scores ranging from 0 to 4 or 8 according to the item: elevated mood (score 0 = absent to 4 = euphoric), increased motor activity (score 0 = absent to 4 = motor excitement), sexual interest (score 0 = normal to 4 = overt sexual acts), sleep (score 0 = no decrease in sleep to 4 = denies need for sleep), irritability (score 0 = absent to 8 = hostile, uncooperative), speech (score 0 = no increase to 8 = pressured, uninterruptible), thought disorder/language (score 0 = absent to 4 = incoherent), content (score 0 = normal to 8 = delusions, hallucinations), disruptive-aggressive behavior (score 0 = absent, cooperative to 8 = assaultive, destructive), appearance (score 0 = appropriate to 4 = completely unkempt, decorated, bizarre garb), and insight (score 0 = present to 4 = denies any behavior changes). The overall score ranges from 0 to 60.

There is currently no explicit definition for residual symptoms or symptomatic remission in bipolar disorder and past studies have used different cut-offs in this patient group. The International Society for Bipolar Disorder (ISBD) Task Force (32) for example, suggested a YMRS score of <8 or >5 to define residual manic symptoms and a MADRS score of 8–14 to define residual depressive symptoms. MADRS-Scores of ≤ 5 or ≤ 7, and YMRS Scores of <8 or <5 have been recommended to define symptomatic remission (32, 33). Based on our previous studies [e.g., (34)], in the current study we used a score of ≤ 8 on both the MADRS and the YMRS to define residual mood symptoms.

### Social Functioning

Social functioning was measured by the Personal and Social Performance (PSP) Scale (35). It is a 100 point single item rating scale which is subdivided into 10 equal intervals, and ratings are based on the assessment of the patients' functioning measured in four domains: (i) socially useful activities, (ii) personal and social relationships, (iii) self-care, and (iv) disturbing and aggressive behavior.

### Self-Stigma/Stigma Resistance

Self-stigma/stigma resistance was assessed by the Internalized Stigma of Mental Illness (ISMI) scale (36), consisting of 29 items with a Likert-scale from 1 = strongly disagree to 4 = strongly agree. The scale itself is composed of five subscales: alienation (6 items), stereotype endorsement (7 items), discrimination experience (5 items), social withdrawal (6 items), and stigma resistance (5 items). The five “stigma resistance” items are reverse-coded and serve as a validity check.

Stigma resistance is theoretically (36) and psychometrically (37) distinct from self-stigma and the current study therefore measured *stigma resistance* using the Stigma Resistance subscale and measured *self-stigma* by summing the averages of the remaining four subscales of the ISMI. The extent of self-stigma/stigma resistance has previously been defined using a cut-off point of 2.5 on the mean item scores (23, 37, 38). Accordingly, a value of 2.5 and above can be applied to define moderate to high self-stigma/stigma resistance and lower than 2.5 for low self-stigma/stigma resistance (38).

### Resilience

Resilience was measured using the Resilience Scale (RS-25) (39), which was the only resilience scale validated in German language at the time of study conduction. The authors of the RS-25 conceptualized resilience as “a positive personality characteristic that enhances individual adaption” (39). It consists of 25 items and is divided into two categories: “acceptance of self and life” (8 items) and “personal competence” (17 items). The subscale “acceptance of self and life” highlights features such as adaptability, tolerance, flexibility, and balance, whereas the subscale “personal competence” summarizes features such as self-reliance, independence, determination, mastery, perseverance, invincibility, and resourcefulness. Since the 2-factor structure could not be identified in the German version (40), we considered only the total score for our study. All items are scored on a 7-score item scale, ranging from 1 = strongly disagree to 7 = strongly agree with possible scores ranging from 25 to 175. Higher scores indicate higher resilience, population-representative norm values are available (133.8 ± 22.5) (40). Schumacher and coworkers reported that age and sex differences are small and therefore hardly of practical importance (40). The developers of the original scale categorized the overall RS-25 score into 3 levels: scores below 125 reflect low resilience, scores between 126 and 145 indicate moderately low to moderate levels of resilience, and scores of 146 and higher indicate high resilience (41).

### Premorbid Functioning

Premorbid functioning was assessed retrospectively using the Cannon-Spoor Premorbid Adjustment Scale (PAS) (42). The PAS was designed to measure “*the degree of success in attainment of certain developmental goals at each phase of a subject's life*” (42). This instrument is divided into a general section and measures two discrete areas of premorbid functioning, academic and social functioning, at each of four developmental stages: childhood (up to age 11), early adolescence (age 12–15), late adolescence (age 16–18), and adulthood (age 19 and older). Since there are concerns regarding the validity of the general section (43), this was section was left out.

Items are scored on a scale from 0 (normal adjustment) to 6 (severe impairment). The range of scoring for each developmental period is the same, allowing for comparison of scores across developmental periods. As a matter of course, adulthood was not assessed in patients with illness onset prior to or at 19 years of age.

### Statistical Methods

All statistical analyses were performed using SPSS, version 26. Statistical testing was done at a 0.05 level of significance. Associations of premorbid functioning, psychopathology, and resilience with self-stigma/stigma resistance were evaluated by means of Spearman rank correlation coefficients, as the majority of the variables involved showed considerable departures from a normal distribution.

The combined effect of patient characteristics (age, sex, education, duration of illness, and a history of psychotic symptoms), premorbid functioning, psychopathology, and resilience on self-stigma/stigma resistance was examined by multiple linear regression analysis. We used backward stepwise variable elimination for the identification of significant predictors. To reduce the number of variables tested, only those variables were entered into the model that had yielded a *p* < 0.1 in the correlation analysis. For control purposes, we ran each regression analysis a second time with forward variable selection, giving rise to the same final model in all cases. As a measure of determination of the regression model, *R*^2^ is reported.

### Power Analysis

The subsequent power analysis was conducted using G^*^Power, version 3.1.7. The sample size of 60 bipolar patients is sufficient to detect, under standard conditions regarding type-one error (two-tailed α = 0.05) and power (1-β = 0.8), Pearson correlations of r = 0.35 or greater. The same applies for Spearman rank correlations. Moreover, under the same conditions regarding α and β, the sample size of 60 allows detection of an effect size of f^2^ = 0.136 in a linear regression analysis with up to 10 independent variables, when testing for the effect of one additional predictor. Both (r = 0.35, and f^2^ = 0.136) are medium effects according to Cohen's classification (44).

## Results

### Patient Characteristics

Demographic and clinical characteristics of the 60 patients we recruited are summarized in Table 1. According to the categorization by Wagnild and Young (39), the sample showed a moderately low to moderate RS-25 mean score (129.8 ± 23.1). In turn, they had a relatively high stigma resistance mean score of 2.87 ± 0.55, whereas the self-stigma mean score (1.86 ± 0.61) was relatively low.

**Table 1:** Patient characteristics (*N* = 60 bipolar patients).

**Variable**	**Mean ± SD or *N* (%)**
Age	43.2 ± 11.0
**Sex**	
Male	25 (41.7%)
Female	35 (58.3%)
Education (years)	13.8 ± 3.2
**Marital status**	
Single	20 (33.3%)
Married/stable partnership	28 (46.7%)
Divorced	12 (20.0%)
**Living situation**	
With original family	5 (8.3%)
With own family	33 (55.0%)
Alone	17 (28.3%)
Others	5 (8.3%)
**Employment**	
Full-time employment	11 (18.3%)
Part-time employment	19 (31.7%)
Supported employment	1 (1.7%)
Training	2 (3.3%)
Retired	14 (23.3%)
Unemployed/Sick-leave	13 (21.7%)
Duration of illness (years)	11.1 ± 10.3
MADRS total score	7.41 ± 8.23
YMRS total score	1.44 ± 2.84
History of psychotic symptoms	9 (15%)
PSP	70 ± 21.5
**Psychotropic medication**	
Antipsychotic	39 (65.0%)
Mood stabilizer	41 (68.3%)
Antidepressant	25 (42.7%)
Benzodiazepine	9 (15.0%)

*SD, Standard deviation; MADRS, Montgomery-Asberg Depression Rating Scale; YMRS, Young Mania Rating Scale; PSP, Personal and Social Performance Scale*.

### Premorbid Functioning

Premorbid academic functioning, as measured by the PAS and shown in Table 2, deteriorated significantly from childhood (0.92 ± 0.86) to early adolescence (1.53 ± 1.22, *p* < 0.001) and late adolescence (1.68 ± 1.26, *p* < 0.001). No significant changes in premorbid social functioning could be found.

**Table 2:** Premorbid functioning as measured by the PAS.

	**Mean**	**SD**
Premorbid functioning (PAS scales, range 0–6)^a^		
PAS Childhood academic functioning	0.92	0.86
PAS Childhood social functioning	1.32	1.52
PAS Early adolescence academic functioning^b^	1.53	1.22
PAS Early adolescence social functioning	1.21	1.19
PAS Late adolescence academic functioning^c^	1.68	1.26
PAS Late adolescence social functioning	0.99	1.10
PAS total score academic functioning	1.37	0.83
PAS total score social functioning	1.18	1.12

*PAS, Premorbid Adjustment Scale*.

a *0, normal functioning; 6, poorest category*.

b *Significantly poorer academic functioning compared to childhood (Z = 3.64, p < 0.001)*.

c *Significantly poorer academic functioning compared to childhood (Z = 3.49, p < 0.001)*.

### Correlation Between Premorbid Functioning and Self-Stigma/Stigma Resistance

There were no significant correlations between premorbid functioning and stigma for any PAS or ISMI subscales (|r| < 0.15 and *p* > 0.2 for all subscales).

### Correlation Between Psychopathology and Self-Stigma/Stigma Resistance

Residual depressive symptoms correlated positively with self-stigma (r = 0.497, *p* = < 0.001) and negatively with stigma resistance (r = −0.373, *p* = 0.003). No significant correlations could be found between residual manic symptoms and self-stigma/stigma resistance. A history of psychotic symptoms did not correlate significantly with self-stigma or stigma resistance.

### Correlation Between Resilience and Self-Stigma/Stigma Resistance

Resilience correlated negatively with self-stigma (r = −0.626, *p* < 0.001) and positively with stigma resistance (r = 0.613, *p* < 0.001) showing that high resilience is associated with low self-stigma and higher stigma resistance.

### Prediction of Self-Stigma/Stigma Resistance by Resilience, Premorbid Functioning, and Sociodemographic and Clinical Data: Results of Multiple Regression Analysis

The combined effect of resilience, premorbid functioning, sociodemographic data and clinical variables (duration of illness, MADRS, YMRS, history of psychotic symptoms) on self-stigma/stigma resistance was investigated by multiple regression analysis (see Table 3). Only resilience emerged as a significant predictor for self-stigma and stigma resistance. Low resilience was a predictor for higher self-stigma and high resilience for higher stigma resistance.

**Table 3:** Results of multiple linear regression analysis.

**Dependent variable**	**Model information Independent variables^**a**^**	**Standardized Beta**	**F**	**d.f. (df1,df2)**	***p*-value**	***R*^2^ adjusted**
Self-stigma	Final model		46.30	1,57	<0.001	0.439
	RS-25 total score	−0.669	46.30	1,57	<0.001	
Stigma resistance	Final model		48.85	1,57	<0.001	0.452
	RS-25 total score	0.506	48.85	1,57	<0.001	

*RS-25, Resilience Scale*.

a *Only those independent variables are shown which were retained in the final model, i.e., statistically significant (p < 0.05) or nearly significant (p < 0.10) predictors*.

*The following independent variables were tested: PAS academic and social functioning, age, sex, education, and clinical variables (duration of illness, MADRS, YMRS, history of psychotic symptoms)*.

## Discussion

In general, factors protecting from self-stigma and promoting stigma resistance in patients suffering from mental illnesses may be integrated in preventive and therapeutic interventions. Previous studies have investigated factors related to self-stigma in patients with bipolar disorder (8, 9, 12, 13, 45), however, this is the first study to examine the relevance of premorbid functioning and resilience in this regard. As sociodemographic data and MADRS and YMRS scores show, we recruited a group of chronically ill patients with only mild symptoms and mild difficulties in social functioning. This approach targets a group of patients one is interested in when evaluating long-term management.

About half of the patients were married or living in a stable partnership, which is in line with earlier studies from other countries (46, 47). On the other hand, around 30–50% of patients considered as clinically remitted have previously been shown to fail to regain premorbid psychosocial functioning, which decreases their capability to take part in normal working life (48). Accordingly, only around half of participants had a full-time or part-time job at the time of study inclusion, whereas approximately one quarter was unemployed or on sick-leave. This points to the chronicity and severity of BD and corroborates the findings of past studies, which have even shown much higher unemployment rates (46, 49, 50). Accordingly, bipolar patients are in need for continuous social support, even after remission is achieved.

Compared to previous studies, as shown in a systematic review by Ellison et al. (51) where patients reported a moderate to high degree of self-stigma, our patients showed relatively low self-stigma and high stigma resistance mean scores. This may be explained by our sample of chronically ill patients which may have developed coping strategies over the years. Considering the above mentioned fact that around half of the study participants were either married or in stable relationships, were living with their own families and were employed, one can hypothesize that these factors also play a substantial role in building stigma resistance. However, this issue cannot be addressed by our data.

With regards to resilience, a below-average RS-25 mean score compared to a norming sample from Germany (40) was recorded, which corroborates our findings in patients suffering from schizophrenia (23) or major depressive disorder (52). We previously found resilience to be associated with bipolar patients' self-esteem (19) and QoL (14). Accordingly, our finding of higher resilience being associated with lower self-stigma and higher stigma resistance was not unexpected. Moreover, multiple linear regression analysis showed resilience to be a positive predictor for stigma resistance and a negative predictor for self-stigma. This suggests that resilience may potentially play a preventive role in the development of self-stigma and may promote stigma resistance and could be an important component of anti-stigma interventions. This should be investigated in future studies. Previously developed anti-stigma interventions with focus on self-stigma, analyzed in a review by Yanos et al. (53), have already used psychoeducation and most of them have integrated cognitive techniques with promising results. However, the authors argued that implementation and outcome are still in their early stages. Accordingly, the long-term role of resilience in bipolar disorder on outcome and the effect of resiliency training programs remain unclear and should also be investigated in future longitudinal studies.

With regards to symptoms, similar to past studies, we found a positive association between depressed mood and self-stigma (54, 55), whereas no association was found between residual manic symptoms and self-stigma (45, 55). However, due to the very low YMRS mean, the latter needs to be interpreted with caution. Nevertheless, past studies have shown that although patients are considered as remitted or euthymic, they still experience low grades of mood symptoms of either depression or mania which might have a negative impact on outcome (34).

Considering that we still found an association between self-stigma/stigma resistance and very mild depressive symptoms (whereby other factors not considered in the current study may also have played a role here), our findings indicate that pharmaco-, psycho-, and sociotherapeutical measures should be exhausted to promote stigma resistance.

The findings of previous studies regarding premorbid functioning of patients developing BD in the course of life are conflicting and its relevance therefore remains unclear. In our sample, patients generally had low PAS scores (indicating high premorbid functioning), similarly as found in past studies (26, 56). We found a deterioration of premorbid academic functioning from childhood to adolescence, whereby premorbid social functioning was stable at all developmental epochs, which corroborates the findings of Paya et al. (57). By contrast, Cannon et al. (24) reported poorer social functioning during adolescence, whereas academic functioning remained stable. These inconsistent findings may be linked to the heterogeneity of samples and different methods used to measure premorbid functioning such as using the PAS-Scale [e.g., (28, 57)], a modified version of the PAS [e.g., (24, 27)], or using school reports and parental interviews [e.g., (56)]. When interpreting the PAS, the presence of symptoms at the time of the interview may also be considered. However, also in this regard there are no consistent findings: Goldberg and Ernst (58) found depressed mood to be related to the PAS score in the adolescence period, whereas Uzelac et al. (27) found it to be related to the childhood period. It has also been discussed that the PAS may not capture important features of BD as it was originally designed for patients with schizophrenia (28). In the current study, we could not find any significant correlations between premorbid functioning and self-stigma or stigma resistance. The generally low PAS scores in our sample may explain this finding and the above discussed points must also be taken into consideration. Therefore, it remains unclear whether premorbid functioning has an impact on self-stigma/stigma resistance in BD. This calls for further studies.

Despite the implications of our findings, the cross-sectional design and the relatively low sample size limit the generalizability of our results. Patients having acute or more severe symptoms or those at an earlier stage of illness may have different levels of self-stigma/stigma resistance and resilience and these may change over the course of time. Clearly, to investigate both the process character of these factors and a potential causal interrelationship, longitudinal studies are necessary. With regards to premorbid functioning, recall bias may have played a role as we did not gather information from family members to support patients' claim. Furthermore, the RS-25 scale only captures personal traits, while resilience is seen as a dynamic process. Again, this may limit the generalizability of our findings.

Lastly, it will be critical to investigate whether other issues not considered in the current study and likely to have an influence on both resilience and self-stigma/stigma resistance, e.g., social support, are of relevance in this context.

Altogether, our findings suggest that resilience might play an important role in preventing self-stigma and building stigma resistance and hence should be considered as a component of self-stigma reduction interventions. Irrespective of these considerations, further efforts are necessary to reduce public stigma toward the mentally ill.

## Data Availability Statement

The original contributions presented in the study are included in the article/supplementary material, further inquiries can be directed to the corresponding author/s.

## Ethics Statement

The studies involving human participants were reviewed and approved by Ethics Committee of the Medical University of Innsbruck, Austria. The patients/participants provided their written informed consent to participate in this study.

## Author Contributions

FP and AH: drafting the article. GK, AH, and FP: data analysis. FP, MB, SP, and BF-A: data collection. All authors study design, approval of the final version, and critical revision of the article.

## Conflict of Interest

The authors declare that the research was conducted in the absence of any commercial or financial relationships that could be construed as a potential conflict of interest.
